# Obesity downregulates lipid metabolism genes in first trimester placenta

**DOI:** 10.1038/s41598-022-24040-9

**Published:** 2022-11-12

**Authors:** Aisha Rasool, Taysir Mahmoud, Begum Mathyk, Tomoko Kaneko-Tarui, Danielle Roncari, Katharine O. White, Perrie O’Tierney-Ginn

**Affiliations:** 1 Tufts Medical Center, Mother Infant Research Institute, Box# 394, 800 Washington Street, Boston, MA 02111 USA; 2 Brandon Regional Hospital, Brandon, FL USA; 3 Department of Obstetrics and Gynecology, Tufts University School of Medicine, Boston, MA USA; 4 Department of Obstetrics and Gynecology, Boston University School of Medicine, Boston, MA USA; 5 Friedman School of Nutrition, Tufts University, Boston, MA USA

**Keywords:** Fat metabolism, Obesity, Reproductive biology

## Abstract

Placentas of obese women have low mitochondrial β-oxidation of fatty acids (FA) and accumulate lipids in late pregnancy. This creates a lipotoxic environment, impairing placental efficiency. We hypothesized that placental FA metabolism is impaired in women with obesity from early pregnancy. We assessed expression of key regulators of FA metabolism in first trimester placentas of lean and obese women. Maternal fasting triglyceride and insulin levels were measured in plasma collected at the time of procedure. Expression of genes associated with FA oxidation (FAO; ACOX1, CPT2, AMPKα), FA uptake (LPL, LIPG, MFSD2A), FA synthesis (ACACA) and storage (PLIN2) were significantly reduced in placentas of obese compared to lean women. This effect was exacerbated in placentas of male fetuses. Placental ACOX1 protein was higher in women with obesity and correlated with maternal circulating triglycerides. The PPARα pathway was enriched for placental genes impacted by obesity, and PPARα antagonism significantly reduced ^3^H-palmitate oxidation in 1^st^ trimester placental explants. These results demonstrate that obesity and hyperlipidemia impact placental FA metabolism as early as 7 weeks of pregnancy.

## Introduction

Pre-pregnancy obesity (body mass index (BMI) > 30 kg/m^2^) impacts > 20% of women in the U.S.^1^, and is associated with both increased short-term health risks during gestation and perinatally to both baby and mother (e.g. gestational diabetes, macrosomia), and long-term risks (e.g. development of diabetes, obesity and cardiometabolic disease) throughout the offspring’s life^2^. Though the mechanisms underlying these associations remain poorly understood, they are widely thought to involve changes in placental development and function.

The placenta controls maternal–fetal nutrient transfer, and produces hormones that modulate maternal metabolic adaptations to pregnancy, impacting nutrient supply and ultimately fetal nutrient delivery and growth^3^, and is thus a key player in fetal development and long-term outcomes. Maternal obesity is associated with impaired placental function at term such as lipid accumulation, impairments in mitochondrial function, inflammation, and oxidative stress^4–8^. This lipotoxic milieu may be initiated by changes in lipid metabolism pathways^6,9^. Fatty acids, oxidized even in the presence of glucose^10^, are a vital source of energy for the placenta, which expresses all enzymes necessary for fatty acid *β*-oxidation (FAO)^11^. Placental FAO is important for development of the fetal-placental unit and health of the mother; deficits in placental FAO have been shown to induce production of cytotoxic metabolites leading to maternal liver damage, and placental insufficiency resulting in fetal growth restriction and prematurity^11^. In placentas of women with obesity, we have previously reported that mitochondrial FAO is lower and lipid storage, likely secondary to elevated fatty acid esterification (FAE) rates, is higher in comparison to lean women late in pregnancy^12^. Placentas of obese women at term also have reduced FAO gene expression including PPARα, a major modulator of FAO, fewer mitochondria and a lower concentration of acylcarnitines which suggest reduced FAO capacity^12^. Changes in lipid metabolism have been shown to alter fatty acid delivery to the fetus^13^, suggesting a fundamental role in both lipotoxicity and fetal growth outcomes. Alterations in FA transporter proteins (FATPs) and FA binding proteins (FABPs) have also been reported in term placentas of obese women^14,15^ although the impact of this on fetal development is unclear.

Currently, most interventions to improve outcomes in pregnancies complicated by obesity begin during the second trimester or later, when placental function is well established, and have only weakly impacted fetal outcomes^16^. This suggests that impacts of obesity begin very early in pregnancy. Indeed, microarray analysis of first trimester placentas showed that obesity is associated with a downregulation of clusters of metabolic genes, including lipid metabolism and mitochondrial pathways^17^. However, a targeted analysis of lipid metabolism pathways and activity in first trimester placentas has not been explored.

To improve therapeutic interventions in pregnancy, the mechanisms by which obesity impacts early placental development must be understood. To test the hypothesis that lipid metabolism is altered in obese placentas beginning in the first trimester, we assessed the following: (1) gene expression of key lipid metabolism modulators and their association with maternal metabolic markers, fetal sex, and gestational age, and (2) effect of inhibition of the major FAO regulator, PPARα, on [^3^H]-palmitate oxidation and esterification in explants isolated from first trimester placentas.

## Methods

### Sample collection

All methods were carried out in accordance with relevant guidelines and regulations. All experimental protocols were approved by MetroHealth Medical Center IRB committee (Cleveland, OH), Boston Medical Center IRB Committee (Boston, MA), and Tufts Medical Center IRB Committee (Boston, MA). Written and informed consent was obtained prior to study participation. Maternal clinical data (including BMI, gestational age via ultrasound, maternal age, smoking status and race/ethnicity) were collected at time of consent from women who had previously chosen to terminate their pregnancy. Exclusion criteria included known fetal anomalies, multiple gestations, hypertension, pre-existing diabetes, substance abuse, or other comorbid diseases that could impact metabolism.

Placental tissue, fasting maternal blood samples, and maternal health data were collected at the time of procedure. Maternal plasma was collected in EDTA-coated tubes, chilled and centrifuged at 1500*g* for 10 min before aliquoting and storing at − 80 °C. Plasma glucose was measured by using hexokinase/G-6-PDH method^18^ and plasma triglycerides were measured using the glycerol phosphate oxidase method^19^. Both were measured using an Abbot Architect c8000 clinical analyzer (spectrophotometer). Plasma insulin was measured using human insulin enzyme-linked immunoassay (ELISA) kits (Crystal Chem). Placental tissue was collected immediately after procedure and rinsed in PBS to remove blood. Decidua was removed and all available villi collected for use in these experiments. Samples for gene expression and protein analysis were blotted, snap frozen in liquid nitrogen and stored at − 80 °C until assayed.

### Placental gene expression analysis

Total RNA was obtained following homogenization of ~ 50 mg placental tissue in TRIzol reagent (Thermofisher) per the manufacturer’s guidelines. RNA integrity was assessed for each sample by visualizing ribosomal RNA via gel electrophoresis and quantified and assessed for purity using a Nanodrop One^C^ (ThermoScientific) spectrophotometer.

Intact samples with 2 visible bands at 18S and 28S, and whose 260/280 and 260/230 absorbance readings measured above 1.7 were included for gene expression analysis. A custom Nanostring nCounter Elements Panel (NanoString Technologies, WA, USA) was used to assess expression of 35 lipid metabolism genes (Supplemental Table 1) and an additional three housekeeping genes (L19, β-actin and Ywhaz). These genes were analyzed in 32 samples (n = 16 for both lean (BMI 18–25 kg/m^2^) and obese (BMI 30–55 kg/m^2^) women) using the nCounter system, and assays were completed according to manufacturers’ instructions using 140 ng of RNA and a 24-h hybridization at 67 °C. Data were analyzed using the proprietary nSolver software (NanoString Technologies, WA, USA) and normalized to internal controls and housekeeping genes to correct for major sources of error including pipetting errors, instrument scan resolution, batch variations, and sample input variability. Five samples in total (n = 2, lean; n = 3, obese) were excluded from further analysis due to differential clustering as outliers in PCA plot data.

### Sex determination of samples

Sex was determined as described previously^20^. Briefly, 250 ng placental RNA were reverse transcribed to cDNA using SuperScript II Reverse Transcriptase kit (Life Technologies, Carlsbad, CA, USA) with Oligo (dt) following manufacturer’s guidelines and cycling conditions. XIST and DDX3Y gene expression was quantified by RT-qPCR using FAM-labelled TaqMan gene expression assays (Life Technologies, XIST: Hs01079824_m1; DDX3Y: Hs00965254_gH), TaqMan universal PCR master mix (Life Technologies) and the QuantStudio 7 Pro Real Time PCR system (Thermofisher Scientific). The cycling conditions for the real-time PCR were: (1) 95 °C for 10 min; (2) 40 cycles of 95 °C for 20 s, 55 °C for 30 s, and 72 °C for 30 s; and (3) final elongation at 72 °C for 10 min. Samples were assayed in duplicate and Ct values were automatically generated by QuantStudio 7 Pro software (Thermofisher Scientific). A Ct = 40 corresponding to the final RT-qPCR cycle was assigned to samples where no Ct value was obtained. Relative gene expression was calculated as ΔCt (XIST Ct − DDX3Y Ct).

### ACOX1 protein analysis

To detect ACOX1 protein levels in placental tissue, ~ 50 mg tissue was homogenized and sonicated. Protein levels were calculated using a Bradford assay and standardized to 0.4 mg/ml. ACOX1 protein levels were then quantified in this tissue homogenate using a commercially available ACOX1 ELISA kit (LSBio Inc, WA), following manufacturer’s instructions.

### ^3^H-Palmitate metabolism assay

Fresh placental tissue was collected into warm PBS as described above. Placental villous explants were dissected as previously described^12^ in a Biospherix Xvivo Hypoxia Workstation (Biospherix, NY). All sample manipulations and incubation conditions were at 37 °C under 6% O_2_ and 5% CO_2._ Conditions were performed in triplicate. Briefly, placental explants were acclimatized to culture medium supplemented with 10% fetal bovine serum, 1% Penicillin/Streptomycin and 0.2% ascorbic acid for 30 min, and then incubated for 4 h in the presence of 0.56% DMSO (Thermofisher Scientific) vehicle or 100 µM of the PPARα antagonist GW6471 (Cayman Chemicals), in addition to control samples. These conditions were then replicated in the presence of 1.25% fatty acid-free BSA (Sigma-Aldrich), 0.1 mmol/L unlabeled palmitate (Sigma-Aldrich), and 18,500 Bq/mL [^3^H]-palmitate (Moravek Biochemicals, Brea, CA) for a further 18 h. *Fatty Acid Oxidation*: After 22 h, medium was collected, and tritiated water (^3^H^2^O), representing the oxidized palmitate, was determined by the phase equilibration method^21^. Data were calculated as nmol palmitate/mg protein/h and expressed as a percentage of vehicle control. *Fatty Acid Esterification*: After 18 h, placental explants were washed with ice-cold PBS and homogenized in 400 μL high-performance liquid chromatography–grade acetone. Following lipid extraction, radioactivity in a 100 μL aliquot, representing esterified palmitate, was counted on a Beckman LS3801 liquid scintillation counter (Beckman Coulter, Brea, CA). Esterification was calculated as nmol palmitate/mg protein/h and expressed as a percentage of vehicle control. Lactate dehydrogenase (LDH) assay (Thermo Scientific) was used to assess cytotoxicity of DMSO and GW6471.

A subset of 1st trimester placental villous explants were exposed to GW6471 or DMSO vehicle for 24 h (n = 3 in triplicate), flash frozen in liquid nitrogen and stored at − 80 °C. RNA was extracted and analyzed with the Custom Nanostring Elements panel as described above.

### Statistics

Group size (n = 16 per group) was based upon power calculations based on preliminary PCR data (n = 4) showing a reduction of PPARα mRNA expression in first trimester placentas of obese women (0.7 ± 0.15 SD compared to 0.85), a sample number of 16 per BMI group was sufficient to achieve over 80% power to detect a significant (*P* < 0.05) difference between group outcomes.

Data was normalized using NSolver analysis software (Version 4.0.70) including nCounter Advanced Analysis (Version 2.0.115). False discovery rate (Q) was analyzed by using the Benjamini, Krieger and Yekutieli method^22^. Directed global significance scores were calculated to measure the extent to which gene sets containing our genes of interest (‘carnitine metabolism’, ‘fatty acyl-CoA biosynthesis’, ‘generic transcription pathway’, ‘PPARα activates gene expression’, ‘signal transduction’, and ‘transport of small molecules’) were up or down regulated relative to maternal obesity.

The effect of fetal sex among maternal BMI groups was analyzed by two-way ANOVA with Tukey’s multiple comparisons test. Correlations between mRNA expression, maternal plasma measurements, and gestational age were assessed using Spearman’s correlation coefficients (GraphPad Prism version 9). *P* < 0.05 was considered statistically significant. All data are presented as means ± SEMs unless noted otherwise.

## Results

Demographic data for participants are summarized in Table 1. Lean women are defined as having a body mass index (BMI) at the time of the procedure of 18–24.9 kg/m^2^ and obese women were defined as having a BMI of > 30 kg/m^2^. By design, the average BMI was significantly different between adiposity groups (*P* < 0.0001). There were no significant differences in maternal age or gestational age at time of procedure between lean and obese women. Fetal sex, race and smoking status did not differ between groups. Maternal fasting cholesterol and insulin were significantly elevated in women with obesity, while there was a trend for triglycerides to be higher (*P* = 0.056). Glucose was not different between groups. Representative H&E stained micrographs from early gestation placenta are shown (Supplemental Figure) from women with and without obesity. No obvious differences in histomorphology were noted.

**Table 1:** Maternal characteristics of the study population.

Maternal	Lean (n = 14)	Obese (n = 13)	*P* value
Age (year)	26.1 ± 5.5	29.4 ± 7.2	0.4
BMI (kg/m^2^)	21.2 ± 1.9	35.8 ± 6.5	< 0.0001
Gestational Age	9.7 ± 1.7	9.4 ± 1.3	0.8
Fetal sex (M:F)	10:4	5:8	0.08
Smoker (N:Y)	10:4	9:4	0.9
Race	9 Caucasian	5 Caucasian	0.096
	1 African American	6 African American	
	2 Mixed Race	1 Hispanic	
	1 Asian	1 Unknown	
	1 Hispanic		
Cholesterol (mg/dl)	141.8 ± 27.9 (n = 11)	180.3 ± 17.3 (n = 7)	0.004
Triglycerides (mg/dl)	67.3 ± 18.9 (n = 11)	91.1 ± 21.4 (n = 7)	0.056
Glucose (mg/dl)	81.1 ± 8.9 (n = 13)	78.4 ± 8.1 (n = 11)	0.8
Insulin (mU/ml)	3.9 ± 2.2 (n = 13)	8.2 ± 6.8 (n = 13)	0.01

Values are means ± SDs. *P* < 0.05. Mann Whitney Tests were used for all except smokers and fetal sex where chi-squared test was used.

### Reduced lipid metabolism gene expression in placentas of women with obesity

**Figure 1:**
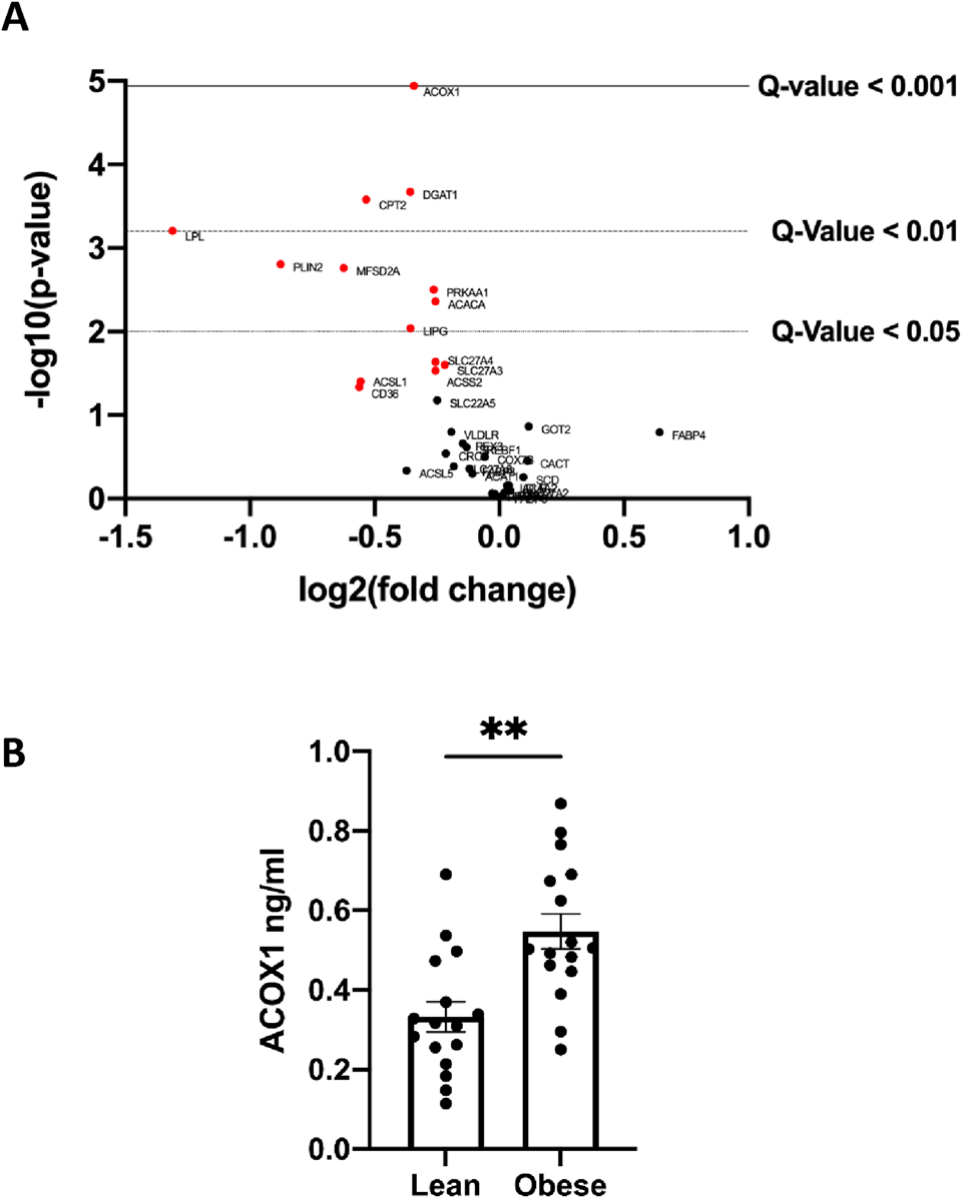
(**A**) Volcano Plot displaying differentially expressed metabolic genes of placentas of obese vs lean women. The vertical axis (y-axis) corresponds to the mean expression value of − log 10 (*P* value), and the horizontal axis (x-axis) displays the log2 fold change value. The red dots represent genes with a *P* value < 0.05 and the horizontal lines represent the q-value significance thresholds. (**B**) ACOX1 protein expression is higher in first trimester placenta of women with obesity vs lean. **P < 0.01 by Students t-test. Data shown is means ± standard error of the mean.

To determine the effect of obesity on placental lipid metabolism in the first trimester placenta, expression of 35 genes was analyzed using the Nanostring Elements platform. Gestational age (GA), smoking, fetal sex, and maternal age were not different between adiposity groups so were not included as confounders. The expression of 13 genes was significantly lower (*P* < 0.05) in placentas from obese women as compared to lean women, and none were higher (Fig. 1A). When false discovery rate via the Benjamini-Krieger-Yekuteli method was assessed, only 8 of these genes were found to be significantly different between groups. These false discovery rate adjusted *P* values are listed as q-value in Supplemental Table 1.

Expression of genes associated with both oxidation and esterification pathways was decreased in placentas of women with obesity. Genes associated with facilitating mitochondrial oxidation (CPT2, PRKAA1) and peroxisomal oxidation (ACOX1), lipases (LPL, LIPG) which hydrolyze triglycerides, FA transporter (MFSD2A), lipid esterification (ACACA, DGAT1), and lipid storage (PLIN2) were lower in first trimester placentas of obese women (Fig. 1A, Supplemental Table 1).

### Increased ACOX1 protein levels in placentas of women with obesity

To determine protein levels of the most downregulated gene by obesity, ACOX1, ELISA was performed on all placentas. The expression of protein was significantly (*P* = 0.0017) increased in placentas from obese woman (Fig. 1B), with no effect of gestational age or sex on this outcome. ACOX1 protein expression correlated with maternal triglyceride levels Spearman R = 0.49, *P* = 0.04 (N = 18).

### PPARα pathway most highly impacted by obesity in first trimester placenta

We calculated directed global significance scores to determine placental lipid metabolism pathways most affected by obesity among the genes analyzed (Table 2). PPARα pathway had the highest scores followed by carnitine metabolism. Multiple downstream targets of PPARα were downregulated although placental PPARα was not detectably impacted itself during the first trimester, unlike at term^12^.

**Table 2:** Directed global significance scores table.

	Directed group: differential expression in obese vs. baseline of lean
PPARA activates gene expression	− 2.978
Carnitine metabolism	− 2.472
Transport of small molecules	− 2.149
Fatty acyl-CoA biosynthesis	− 2.018
Signal transduction	− 1.731
Generic transcription pathway	− 1.488

### PPARα antagonism decreases fatty acid oxidation but not esterification in first trimester placental explants

**Figure 2:**
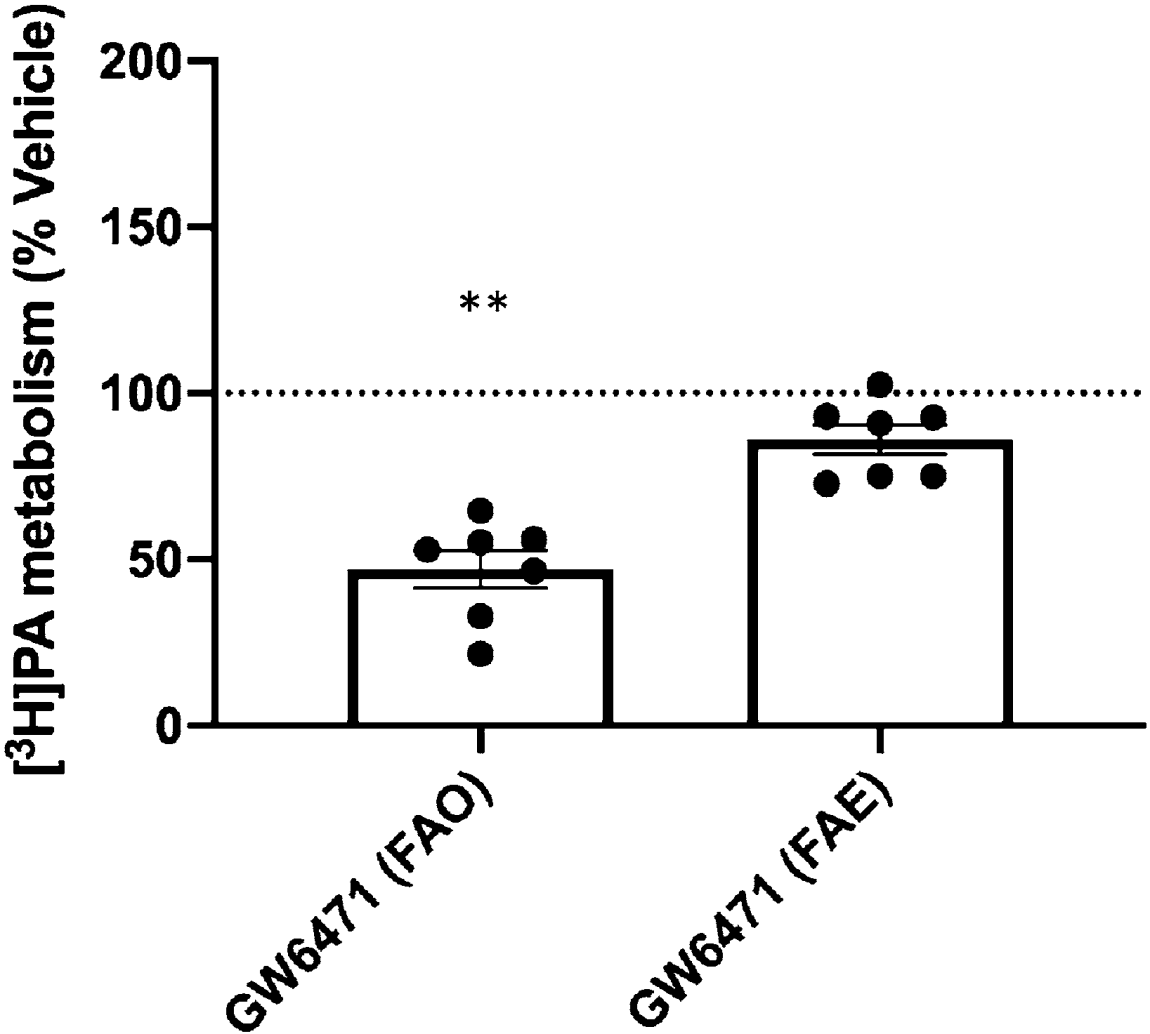
GW6471 reduces fatty acid oxidation but not esterification at 100 µM concentration in first trimester placental explants. **P = 0.008. Vehicle and control treated explants were not different. Data are calculated as nmol palmitate/mg protein/h and expressed as percentage relative to vehicle. Data shown as means ± standard error of the mean.

**Figure 3:**
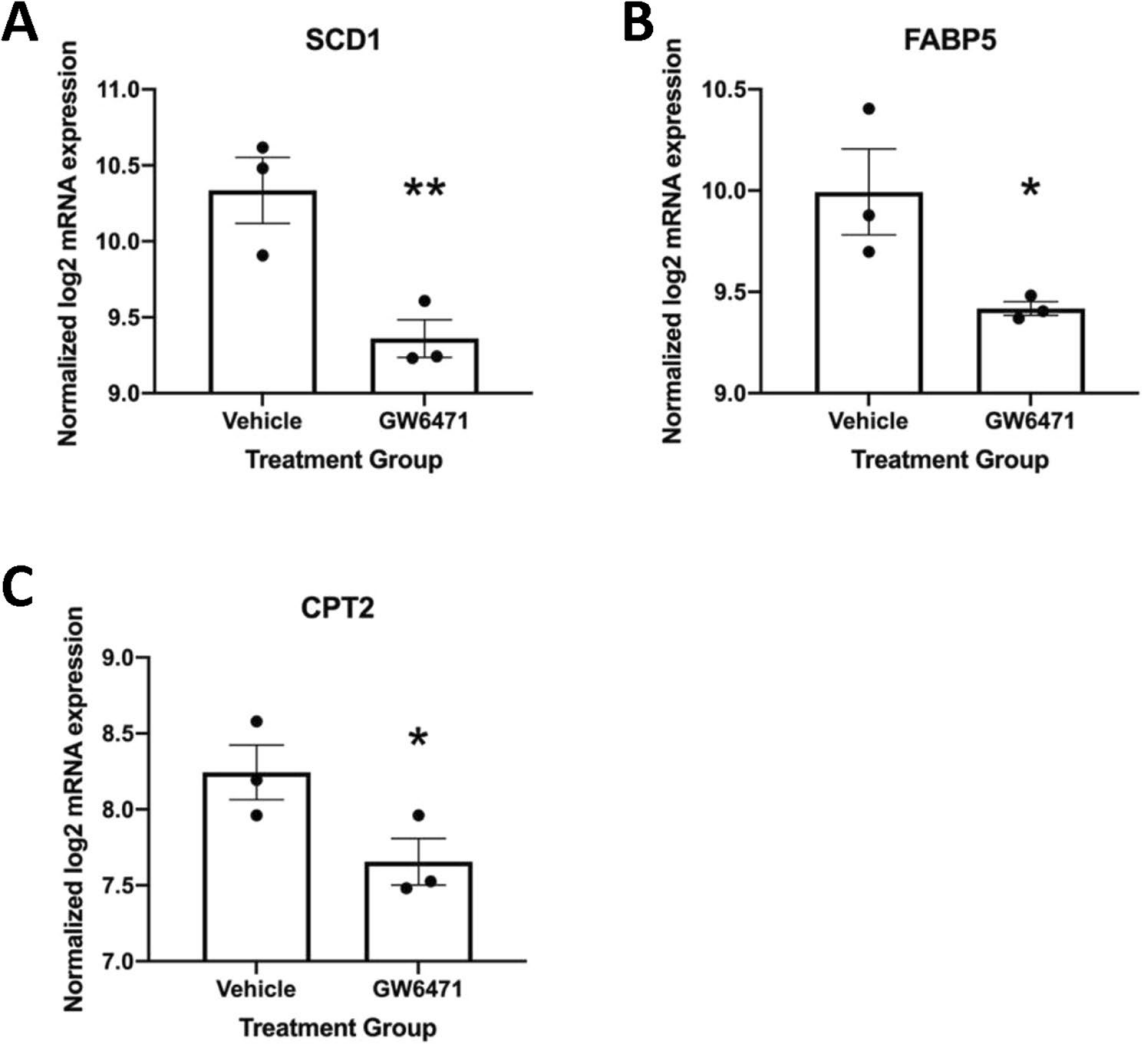
GW6471 (100 µM for 24 h) reduces expression of SCD1 (**A**), FABP5 (**B**) and CPT2 (**C**) genes in first trimester placental explants. **P < 0.01; *P < 0.05. Data shown as means ± standard error of the mean.

To determine the effect of inhibiting the PPARα pathway on downstream placental lipid metabolism, particularly FAO, we used the PPARα antagonist, GW6471. In a separate cohort (maternal characteristics can be found in Supplemental Table 2), we measured ^3^H-palmitate oxidation and esterification rates in first trimester placental explants from healthy (n = 7) women in response to 22 h GW6471 exposure. There was no effect of BMI or GA on GW6471 response, so all results were examined together. Incubation with GW6471 decreased ^3^H-palmitate oxidation by 53% compared with DMSO vehicle alone (*P* = 0.008) in first trimester placental explants (Fig. 2). ^3^H-palmitate esterification rates were not significantly altered by PPARα antagonism. Explants exposed to 100 µM GW6471 showed reduced expression of 3 genes: SCD1 (*P* = 0.008), FABP5 (*P* = 0.028) and CPT2 (*P* = 0.041) (Fig. 3). Neither GW6471 nor DMSO alone induced cytotoxicity in the placental explants based on LDH assay (data not shown).

### Effect of maternal metabolic indicators on placental lipid metabolism gene expression in early pregnancy

To gain insight into the effect of the maternal metabolic profile on mRNA expression in early pregnancy, maternal fasting triglycerides (TG), insulin, and glucose levels were measured at time of procedure. Interestingly, of the genes sensitive to maternal BMI, only ACOX1 and MFSD2A were negatively correlated with TG, and CPT2 was negatively correlated with insulin (Table 3). In addition to these adiposity-sensitive genes, maternal TG negatively correlated with ACSS2, SREBP1, and ACSL5, which are associated with FA synthesis and esterification. Maternal insulin was negatively correlated with DGAT1, the rate-limiting step in TG synthesis, and glucose was negatively correlated with COX7b associated with FA oxidation. This indicates that while these maternal metabolic markers are relevant, there are other factors associated with increased maternal BMI that impact placental lipid metabolism.

**Table 3:** Gene correlations with maternal values.

	Gene	Triglycerides (mg/dl)		Insulin (mg/dl)		Glucose (mg/dl)	
		r	*P*	r	*P*	r	*P*
**Peroxisomal oxidation**							
ACOX1	ACOX1	− 0.6429	**0.004**	− 0.2609	0.20	− 0.1636	0.45
PEX3	PEX3	− 0.3581	0.14	− 0.3750	0.059	0.0796	0.72
COT	CROT	− 0.0423	0.87	− 0.1207	0.56	0.0727	0.74
**Mitochondrial FA oxidation**							
CPT2	CPT2	− 0.3065	0.22	− 0.4838	**0.012**	0.0296	0.89
AMPKα	PRKAA1	− 0.2384	0.34	− 0.1658	0.42	− 0.0848	0.69
OCTN2	SLC22A5	− 0.3540	0.15	− 0.3224	0.11	− 0.2889	0.17
COX-7b	COX7B	0.1476	0.56	0.0386	0.85	0.4747	**0.019**
CACT	CACT	− 0.0485	0.85	− 0.0523	0.80	− 0.1031	0.63
ACAT-1	ACAT1	− 0.1001	0.69	− 0.1829	0.37	0.1497	0.49
ACAA2	ACAA2	− 0.3581	0.14	0.1234	0.55	− 0.1592	0.46
PPAR α	PPARA	− 0.2178	0.39	− 0.0865	0.67	− 0.1314	0.54
CPT1b	CPT1B	− 0.1187	0.64	− 0.1138	0.58	− 0.1083	0.61
**Lipases**							
LPL	LPL	− 0.4241	0.08	− 0.3210	0.11	0.0044	0.98
EL	LIPG	− 0.2859	0.25	0.0010	0.99	0.3276	0.12
**FA uptake and transporters**							
MFSD2A	MFSD2A	− 0.6058	**0.008**	− 0.2595	0.20	0.1031	0.63
FATP4	SLC27A4	− 0.3127	0.21	− 0.2691	0.18	0.0048	0.98
FATP3	SLC27A3	− 0.1620	0.52	− 0.1371	0.50	− 0.0470	0.83
CD36	CD36	− 0.2735	0.27	− 0.3415	0.0877	0.0287	0.89
FABPpm	GOT2	0.3065	0.22	0.2027	0.32	0.3602	0.084
FABP4	FABP4	− 0.4427	0.066	− 0.1665	0.42	− 0.2689	0.20
VLDL-R	VLDLR	− 0.0114	0.96	− 0.1781	0.38	− 0.1953	0.36
FATP6	SLC27A6	− 0.2054	0.41	0.1535	0.45	− 0.0057	0.98
FABP3	FABP3	− 0.1517	0.55	− 0.0003	0.99	0.1692	0.43
LDL-R	LDLR	− 0.4180	0.08	− 0.0653	0.75	0.1727	0.42
FATP2	SLC27A2	0.1930	0.44	− 0.0113	0.96	0.3337	0.11
FABP5	FABP5	− 0.1703	0.50	− 0.0516	0.80	− 0.2593	0.22
**Lipogenesis**							
PLIN2	PLIN2	− 0.0485	0.85	− 0.2827	0.16	0.0844	0.70
ACC	ACACA	− 0.4592	0.055	− 0.1726	0.40	0.1149	0.59
ACSS2	ACSS2	− 0.6347	**0.005**	− 0.3149	0.12	0.1497	0.49
ACSL1	ACSL1	− 0.3478	0.16	− 0.1685	0.41	0.0605	0.78
ACSL5	ACSL5	− 0.5005	**0.034**	− 0.0879	0.67	− 0.1253	0.56
SREBP1	SREBF1	− 0.6099	**0.007**	− 0.1856	0.36	− 0.1344	0.53
SCD-1	SCD	− 0.0196	0.94	0.2062	0.31	0.3115	0.14
PPARγ	PPARG	− 0.1228	0.63	− 0.1644	0.42	0.0087	0.98
DGAT1	DGAT1	− 0.4191	0.095	− 0.5608	**0.0035**	− 0.0445	0.84

Data correlated by Spearmans rank.

Significant values are in bold.

### Gene expression differences in placentas of male and female fetuses

We assessed the effects of maternal obesity on placental gene expression in male vs female fetuses separately due to known sex-specific effects of the maternal environment on the placenta. To test potential interactions between sex and BMI, we used two-way ANOVA. There was no interaction or overall effect of sex in this cohort, but there was a significant effect of BMI on placental ACOX1 (*P* < 0.0001), CPT2 (*P* = 0.0007), LPL (*P* = 0.007), ACACA (*P* = 0.013), PLIN2 (*P* = 0.01), MFSD2A (*P* = 0.006), AMPKα (*P* = 0.009) and EL (*P* = 0.028) consistent with previous analyses (Suppl Table 1). There was also an effect of BMI on OCTN2 (*P* = 0.035) and FATP4 (*P* = 0.035) once fetal sex was accounted for.

**Figure 4:**
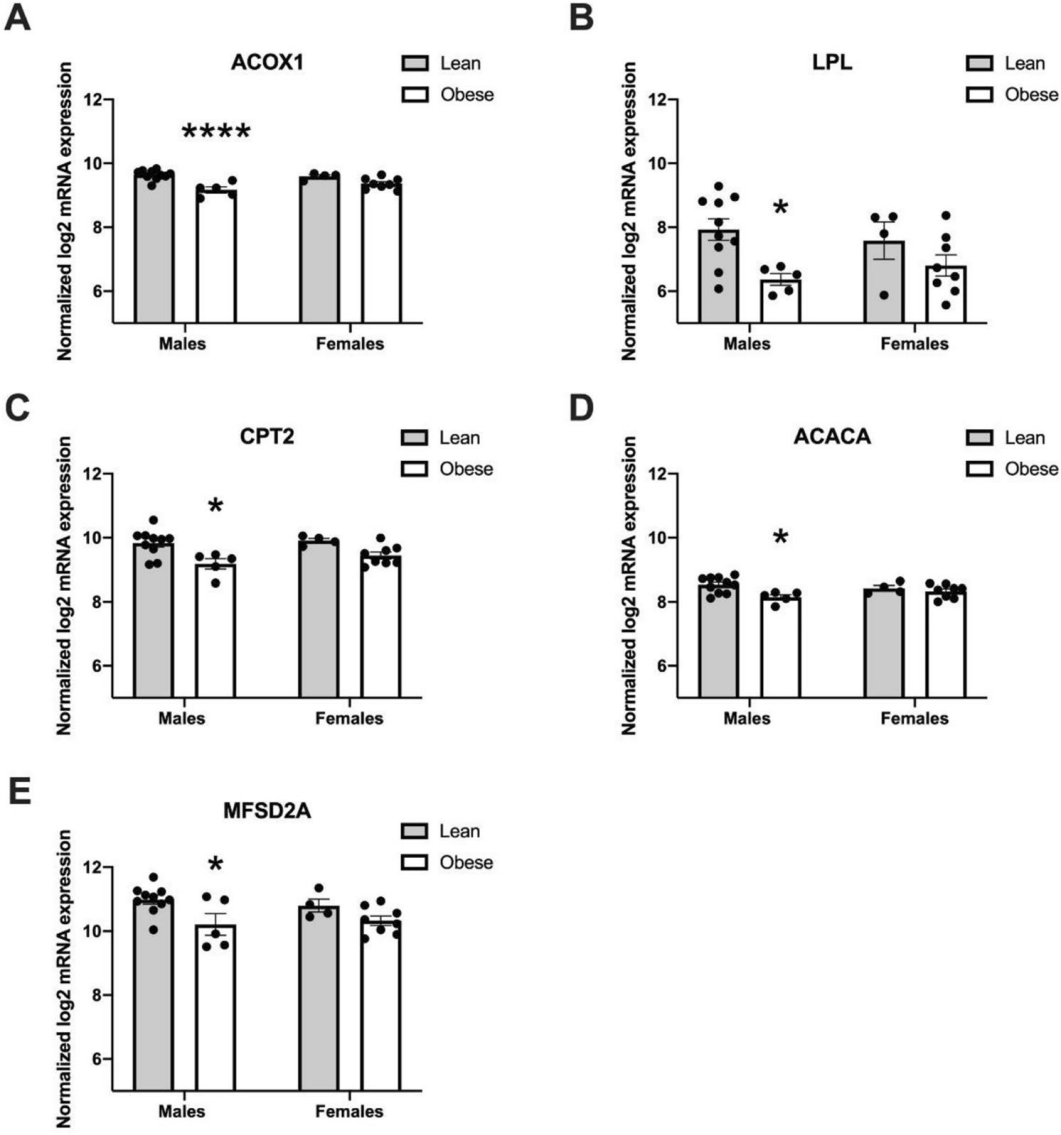
Male fetal sex drives downregulation of fatty acid metabolism associated genes (**A** ACOX1; **B** LPL; **C** CPT2; **D** ACACA; **E** MFSD2A) in women with obesity (Males; lean n = 10, obese n = 5, Females; lean n = 4, obese n = 9). Data (means ± standard error of the mean) are expressed as normalized log2 mRNA expression. *P < 0.05; ***P = 0.0002, data compared by 2-way ANOVA.

Post hoc analysis revealed that male fetuses were the most sensitive to maternal BMI such that when separated by sex, mRNA expression of ACOX1 (*P* = 0.0002), LPL (*P* = 0.03), CPT2 (*P* = 0.01), ACACA (*P* = 0.02) and MFSD2A (*P* = 0.04) were significantly reduced in placentas of obese women with male fetuses but not females (Fig. 4). PLIN2 also tended to be reduced (*P* = 0.052) in this same group. There were no statistical differences between GA, maternal age, smoking or race between BMI groups among sexes (Supplemental Table 3).

### Effect of gestational age on placental lipid transporters and lipases

**Figure 5:**
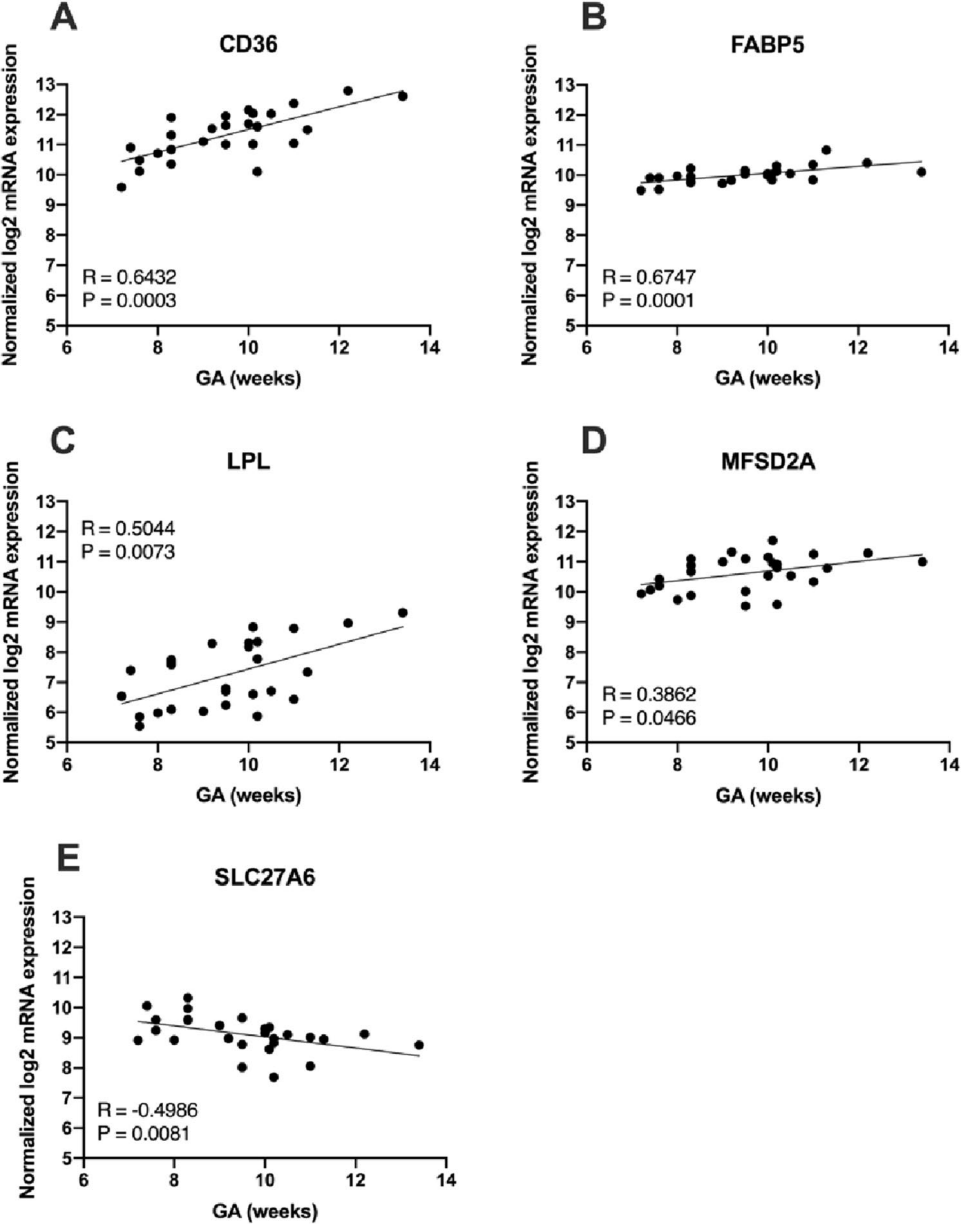
mRNA expression of fatty acid metabolism genes with increasing GA. (**A**) CD36, (**B**) FABP5, (**C**) LPL (**D**) MFSD2A increase significantly with GA and (**D**) SLC27A6 decreases with GA.

The effect of GA on placental lipid metabolism gene expression in the first trimester was also assessed. As the effect of GA was not different between adiposity groups, all participants were analyzed together. Expression of placental genes associated with FA transport (CD36, FABP5, MFSD2A and LPL) were positively correlated with increasing GA (Fig. 5). Only one FA transporter, SLC27A6, negatively correlated with GA.

## Discussion

The key finding of this study was that maternal obesity impacts placental lipid metabolism from the first trimester of pregnancy and that placentas of male fetuses may be more sensitive to these changes compared to female fetuses. All genes we found to be differentially expressed in placentas of obese women were downregulated across fatty acid oxidation, esterification and transport pathways. Evidence that placental lipid metabolism is altered as early as 7 weeks after conception in women with obesity, suggests that early intervention, perhaps even pre-conception, may be necessary to modify and/or improve placental function.

Obesity in early pregnancy is associated with depressed expression of genes in both FA oxidation (FAO) and esterification (FAE) pathways in first trimester placenta. This decrease in expression of lipid metabolism pathways is consistent with previous research assessing the effect of obesity on gene expression in early gestation placenta^17^, particularly the finding of reduced mitochondrial function^23^. Contrary to the effects of obesity in full term placenta^12^, mRNA expression of FAO-related gene CPT2 and FAE-related genes DGAT1 and ACC (ACACA) were downregulated in the first trimester placenta of women with obesity. Although placental PPARα and its targets (CPT1b, PGC1) were downregulated by obesity at term^12^, PPARα expression was not altered in the first trimester placenta. Several other placental genes upregulated by obesity at term (PPARγ, SCD1, CACT, OCTN2, COT) were not significantly altered in early pregnancy, which may indicate a cumulative effect of the obese milieu on placental lipid metabolism pathways throughout gestation. Of note, direct comparisons between these early and late pregnancy studies are limited due to differing methodology (PCR vs Nanostring) used for gene expression analysis.

The cellular energy sensor, AMPKα, is critical for normal placental and embryonic development due to its involvement in cell morphology, growth rate and nutrient transport in trophoblast cells^24^; thus its downregulation in placentas of women with obesity at this gestational stage may be highly impactful. Activation of AMPKα induces fatty acid oxidation pathways via inhibition of ACC, but also potentially via PPARα mRNA upregulation^25^, thereby increasing cellular ATP^26^. The reduction in AMPKα, along with key FAO-regulators downstream of PPARα, such as CPT2 and ACOX1, is consistent with a significant reduction in cellular capacity for both mitochondrial (CPT2^27^) and peroxisomal (ACOX1^28^) FA oxidation in placentas of obese women in early pregnancy. These gene expression changes alone may have implications for cellular energy production and placental efficiency. In addition to impacts on FAO, downregulation of DGAT1 and PLIN2, responsible for triglyceride synthesis and lipid droplet accumulation respectively^29,30^, may alter lipid processing and storage capacity in the early placenta, which could have consequences for fetal FA delivery^13^, though the significance of these changes at this early gestational age is unclear.

Observed lipase and FA transporter expression changes in placentas of women with obesity may reduce placental lipid uptake in the first trimester. Lipoprotein lipase (LPL) and endothelial lipase (EL) hydrolyze triglycerides^31^ and phospholipids, respectively, at the cell membrane, releasing fatty acids for cellular uptake^32^. Placental lipase expression is associated with alterations in fetal growth^33,34^. The downregulation of MFSD2A, a key transporter for the omega-3 (n-3) fatty acid, docosahexaenoic acid (DHA), in first trimester placenta of obese women may be linked to the previously noted decreases in PPARα associated pathways^35^. This reduced placental MFSD2A expression may alter DHA transport to the fetus^36^, which has consequences for fetal neurodevelopment. MFSD2A is also important for trophoblast fusion^37^, and thus is vital for early placental development. Overall, dysregulation of these lipid metabolism genes may lead to fetal growth restriction or pre-eclampsia among other pregnancy-related disorders^38–40^.

Interestingly, despite this overall decrease in gene expression, we detected a significant increase in ACOX1 protein levels in placentas of obese women. This could in part explain the impact we see on the PPARα pathway in these placentas. ACOX1 – the rate limiting step in peroxisomal FA oxidation—metabolizes the endogenous ligands of PPARα, which in turn reduces PPARα activation^41–43^. We previously reported that in term placenta of women with obesity, high peroxisomal FA oxidation compensated for impairments in mitochondrial β-oxidation^12^. Recent studies suggest that high levels of ACOX1 activity is associated with high fat diet and obesity, and can generate reactive oxygen species which impair mitochondrial β-oxidation^42^. ACOX1 activity may be upregulated by the higher plasma lipids^42^ in obese women in early pregnancy, as suggested by the positive correlation with maternal triglyceride levels. Future studies should investigate the interplay between peroxisomal and mitochondrial function in early gestation.

Maternal metabolites commonly associated with obesity (TG, insulin, glucose) did not appear to be overall drivers of the changes in gene expression of placental lipid metabolism pathways in early pregnancy. Although maternal triglycerides were negatively associated with ACOX1 and MFSD2A, and insulin negatively associated with CPT2 and DGAT1, there was no clear identification of the mechanism by which maternal obesity impacts the majority of placental lipid metabolism genes in early pregnancy. Insulin has been shown to down-regulate multiple lipid oxidation and esterification genes in primary first trimester trophoblasts in vitro^17^, though CPT2 and DGAT1 were not among them. However, in vivo maternal hyperinsulinemia did not have the same impact^17^, more consistent with our findings. We speculate that a combination of maternal hyperinsulinemia, lipidemia and potentially unmeasured metabolites interact to create a milieu in vivo which impacts placental lipid metabolism pathways. We recognize that specific types of FA (e.g. LCPUFA, saturated FA) may influence FA transporters and metabolic genes in a FA species-specific manner, but this information was not captured in our study.

We have previously shown that PPARα and mitochondrial ^3^H-palmitate oxidation rates were decreased in full-term placentas of women with obesity^12^. Consistent with this, we found that the PPARα pathway was significantly impacted in first trimester placentas of women with obesity, suggesting that these changes seen at term begin in the early stages of pregnancy. In this study, PPARα antagonism via GW6471 reduced rates of ^3^H-palmitate oxidation in first trimester placental explants by ~ 50%, showing that this pathway is a key regulator of FAO from early pregnancy, supporting findings seen at term. PPARα is also involved in numerous other pathways including oxidative stress and inflammation inhibition^44,45^, thus causing a wide-ranging effect when function is impaired which could contribute to the elevated inflammation observed in placentas of obese women^4^. Interestingly, despite robustly decreasing FAO, PPARα antagonism via GW6471 significantly decreased expression of only one FAO associated gene: CPT2. PPARα expression itself was not significantly reduced. GW6471 acts by displacing coactivators from PPARα. This promotes corepressor binding and is mechanistically linked to the antagonist activity of the molecule^46^. This antagonism thus may not affect PPARα mRNA expression but its transcriptional activity on downstream genes. The PPARα pathway primarily activates FAO, with antagonism having no significant effect on FAE in our study. However, when assessing gene expression, GW6471 exposure reduced SCD1, which is the rate limiting step in the formation of monounsaturated FA. These findings are consistent with previous data showing PPARα may influence lipogenesis by increasing the transcription of SCD1 and other lipogenic genes by regulating the primary transcription factors SREBP1 and liver X receptor α (LXRα)^47,48^. Indeed, as there was a trend for a decrease in esterification with PPARα antagonism, it is possible that a longer exposure to GW6471 or post-exposure period would be required to detect changes in esterification resulting from the observed decreases in SCD1.

Due to previously recognized sexually dimorphic responses to maternal obesity^49,50^, determining the sex of these first trimester placentas was critical to understanding whether sex differences are established in placentas from early pregnancy. We observed that placentas of male fetuses were more sensitive to maternal obesity, which is consistent with existing literature showing impaired placental function at term such as reduced FA uptake, FA transporter and antioxidant levels (which may impact fetal growth and gestational diabetes and preeclampsia risk)^51,52^. Notably, there were no interaction effects between sex and BMI, showing that maternal obesity did not have significantly different impacts in placentas of male and female fetuses in early pregnancy. However, males had a greater overall reduction in 5 of the most significantly affected lipid metabolism genes. These genes: ACOX1, CPT2, ACACA, LPL, MFSD2A have varying functions including fatty acid oxidation, lipogenesis, lipase, and DHA transport, as discussed previously. This exacerbated lipid metabolism gene downregulation in placentas of male fetuses due to maternal obesity could help explain increased incidence of poorer outcomes seen at term , and also long-term outcomes such as altered brain and metabolic development in male offspring compared to females^53,50,54,55^. It has also recently been shown that fetal sex can modify the transcriptome at the maternal-placental interface as early as the first trimester of pregnancy^56^. These sexually dimorphic responses to maternal obesity may be due to male offspring’s higher sensitivity to maternal nutritional cues in utero as part of having a more efficient placenta^57^.

In addition to effects of maternal adiposity, we found that five FA transporters were significantly affected by gestational age, showing that first trimester placental development is a dynamic period. These transporters may have distinct sensitivities to changes in maternal environment depending on gestational age.

Strengths of this study include: targeted analysis of lipid metabolism in first trimester placenta paired with maternal plasma markers of metabolism, functional assays, and measurement of fetal sex. Limitations in the study included only comparing placentas from lean and obese women, instead of analyzing the full range of BMI (such as overweight). BMIs included in the study were also not extreme in the population, so it is unclear how severe obesity and underweight would impact lipid metabolism pathways. This study was also limited by number of samples, and we anticipate increased sensitivity to further reduction in other lipid metabolism genes and ability to detect further sex differences when a larger sample is analyzed. The small amounts of available tissue from first trimester placenta (< 1 g for 7–8 weeks gestation) limited the number of analytes we could assess. Future proteomics analysis of lipid pathways, assessment of fatty acid oxidation rates in early gestation by maternal BMI, and placental hormone production^58^ would improve our understanding of the breadth of functional consequences of obesity so early in placental development.

**Figure 6:**
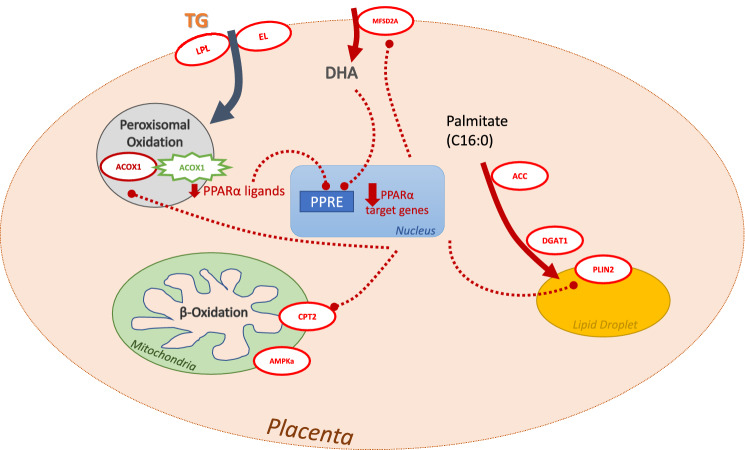
Summary and proposed model of impact of maternal obesity on placental lipid metabolism. High maternal triglycerides (TG) drive fatty acid uptake into the trophoblast, increasing (green) peroxisomal ACOX1 protein (star symbol/shape) activity and oxidation of long chain fatty acids, including PPARα ligands. The decrease in endogenous ligands for PPARα reduces PPAR response element (PPRE) binding and thus decreases (red) the expression of PPARα target genes (e.g. MFSD2a, CPT2, PLIN2, ACOX1 in oval shapes). Low MFSD2a levels may reduce DHA uptake, another PPARα ligand, further decreasing this pathway. Reduction in lipid esterification and storage may lead to accumulation of free FA and lipotoxicity as seen later in gestation. Red dotted lines = inhibition of PPARα target genes Red arrows = inhibition of FA entry to pathway.

In conclusion, this study demonstrates that maternal obesity impacts placental lipid metabolism as early as 7 weeks of gestation. Placentas of male fetuses may be more susceptible to these changes. The downregulation of lipid metabolism genes in early pregnancy from fatty acid oxidation, esterification and synthesis, and lipid transfer pathways, could lead to a less efficient placenta. High placental ACOX1 protein levels may suppress PPARα target gene activation, leading to the downregulation in gene expression (see summary Fig. 6). Placentas from women with FAO disorders are unable to properly oxidize fats, which leads to an accumulation of oxidized metabolites and complications in pregnancy for both mother (e.g. liver disease, preeclampsia) and fetus (e.g. growth restriction and prematurity)^11^. The early placental changes we report highlight the importance of healthy maternal BMI during pregnancy, and that current intervention strategies may need to begin earlier (or even prior to conception) to mitigate impacts of obesity.

## Supplementary Information

Supplementary Information.

## Data Availability

The datasets generated and/or analysed during the current study are available in the Gene Expression Omnibus (GEO) repository, https://www.ncbi.nlm.nih.gov/geo/query/acc.cgi?acc=GSE206037.

## References

[1] Singh GK, DiBari JN (2019). Marked disparities in pre-pregnancy obesity and overweight prevalence among US women by race/ethnicity, nativity/immigrant status, and sociodemographic characteristics, 2012–2014. J. Obes..

[2] Catalano PM, Shankar K (2017). Obesity and pregnancy: Mechanisms of short term and long term adverse consequences for mother and child. BMJ.

[3] Díaz P, Powell TL, Jansson T (2014). The role of placental nutrient sensing in maternal-fetal resource allocation. Biol. Reprod..

[4] Challier JC, Basu S, Bintein T, Minium J, Hotmire K, Catalano PM (2008). Obesity in pregnancy stimulates macrophage accumulation and inflammation in the placenta. Placenta.

[5] Mele J, Muralimanoharan S, Maloyan A, Myatt L (2014). Impaired mitochondrial function in human placenta with increased maternal adiposity. Am. J. Physiol. Endocrinol. Metab..

[6] Saben J, Lindsey F, Zhong Y, Thakali K, Badger TM, Andres A (2014). Maternal obesity is associated with a lipotoxic placental environment. Placenta.

[7] Lewis RM, Desoye G (2017). Placental lipid and fatty acid transfer in maternal overnutrition. Ann. Nutr. Metab..

[8] Hirschmugl B, Desoye G, Catalano P, Klymiuk I, Scharnagl H, Payr S (2017). Maternal obesity modulates intracellular lipid turnover in the human term placenta. Int. J. Obes. (Lond.).

[9] Jarvie E, Hauguel-de-Mouzon S, Nelson SM, Sattar N, Catalano PM, Freeman DJ (2010). Lipotoxicity in obese pregnancy and its potential role in adverse pregnancy outcome and obesity in the offspring. Clin. Sci. (London, England: 1979).

[10] Visiedo F, Bugatto F, Sánchez V, Cózar-Castellano I, Bartha JL, Perdomo G (2013). High glucose levels reduce fatty acid oxidation and increase triglyceride accumulation in human placenta. Am. J. Physiol. Endocrinol. Metab..

[11] Shekhawat P, Bennett MJ, Sadovsky Y, Nelson DM, Rakheja D, Strauss AW (2003). Human placenta metabolizes fatty acids: Implications for fetal fatty acid oxidation disorders and maternal liver diseases. Am. J. Physiol. Endocrinol. Metab..

[12] Calabuig-Navarro V, Haghiac M, Minium J, Glazebrook P, Ranasinghe GC, Hoppel C (2017). Effect of maternal obesity on placental lipid metabolism. Endocrinology.

[13] Perazzolo S, Hirschmugl B, Wadsack C, Desoye G, Lewis RM, Sengers BG (2017). The influence of placental metabolism on fatty acid transfer to the fetus. J. Lipid Res..

[14] Dube E, Gravel A, Martin C, Desparois G, Moussa I, Ethier-Chiasson M (2012). Modulation of fatty acid transport and metabolism by maternal obesity in the human full-term placenta. Biol. Reprod..

[15] Lager S, Ramirez VI, Gaccioli F, Jang B, Jansson T, Powell TL (2016). Protein expression of fatty acid transporter 2 is polarized to the trophoblast basal plasma membrane and increased in placentas from overweight/obese women. Placenta.

[16] Louise J, Poprzeczny AJ, Deussen AR, Vinter C, Tanvig M, Jensen DM (2021). The effects of dietary and lifestyle interventions among pregnant women with overweight or obesity on early childhood outcomes: An individual participant data meta-analysis from randomised trials. BMC Med..

[17] Lassance L, Haghiac M, Leahy P, Basu S, Minium J, Zhou J (2015). Identification of early transcriptome signatures in placenta exposed to insulin and obesity. Am. J. Obstet. Gynecol..

[18] Neeley WE (1972). Simple automated determination of serum or plasma glucose by a hexokinase/glucose-6-phosphate dehydrogenase method. Clin. Chem..

[19] Fossati P, Prencipe L (1982). Serum triglycerides determined colorimetrically with an enzyme that produces hydrogen peroxide. Clin. Chem..

[20] Hoch D, Novakovic B, Cvitic S, Saffery R, Desoye G, Majali-Martinez A (2020). Sex matters: XIST and DDX3Y gene expression as a tool to determine fetal sex in human first trimester placenta. Placenta.

[21] Hughes SD, Quaade C, Johnson JH, Ferber S, Newgard CB (1993). Transfection of AtT-20ins cells with GLUT-2 but not GLUT-1 confers glucose-stimulated insulin secretion. Relationship to glucose metabolism. J. Biol. Chem..

[22] Benjamini Y, Krieger AM, Yekutieli D (2006). Adaptive linear step-up procedures that control the false discovery rate. Biometrika.

[23] Zhou, J., Lassance, L., Minium, J., Catalano, P. & Hauguel deMouzon, S. Obesity modifies placental mitochondrial function in early pregnancy. *Reprod. Sci.* **22**(1 (Suppl 1)), S253 (Abstract) (2015).

[24] Carey EA, Albers RE, Doliboa SR, Hughes M, Wyatt CN, Natale DR (2014). AMPK knockdown in placental trophoblast cells results in altered morphology and function. Stem Cells Dev..

[25] Lee WJ, Kim M, Park HS, Kim HS, Jeon MJ, Oh KS (2006). AMPK activation increases fatty acid oxidation in skeletal muscle by activating PPARalpha and PGC-1. BiochemBiophysResCommun..

[26] Kaufman MR, Brown TL (2016). AMPK and placental progenitor cells. Exp. Suppl..

[27] Barrero MJ, Camarero N, Marrero PF, Haro D (2003). Control of human carnitine palmitoyltransferase II gene transcription by peroxisome proliferator-activated receptor through a partially conserved peroxisome proliferator-responsive element. Biochem. J..

[28] Lake BG (1995). Mechanisms of hepatocarcinogenicity of peroxisome-proliferating drugs and chemicals. Annu. Rev. Pharmacol. Toxicol..

[29] Cases S, Smith SJ, Zheng YW, Myers HM, Lear SR, Sande E (1998). Identification of a gene encoding an acyl CoA:diacylglycerol acyltransferase, a key enzyme in triacylglycerol synthesis. Proc. Natl. Acad. Sci. USA.

[30] Bildirici I, Schaiff WT, Chen B, Morizane M, Oh SY, O'Brien M (2018). PLIN2 is essential for trophoblastic lipid droplet accumulation and cell survival during hypoxia. Endocrinology.

[31] Mead JR, Irvine SA, Ramji DP (2002). Lipoprotein lipase: Structure, function, regulation, and role in disease. J. Mol. Med. (Berl.)..

[32] Khetarpal SA, Vitali C, Levin MG, Klarin D, Park J, Pampana A (2021). Endothelial lipase mediates efficient lipolysis of triglyceride-rich lipoproteins. PLoS Genet..

[33] Gauster M, Hiden U, Blaschitz A, Frank S, Lang U, Alvino G (2007). Dysregulation of placental endothelial lipase and lipoprotein lipase in intrauterine growth-restricted pregnancies. JClinEndocrinolMetab..

[34] Heerwagen MJR, Gumina DL, Hernandez TL, Van Pelt RE, Kramer AW, Janssen RC (2018). Placental lipoprotein lipase activity is positively associated with newborn adiposity. Placenta.

[35] Berger JH, Charron MJ, Silver DL (2012). Major facilitator superfamily domain-containing protein 2a (MFSD2A) has roles in body growth, motor function, and lipid metabolism. PLoS ONE.

[36] Prieto-Sánchez MT, Ruiz-Palacios M, Blanco-Carnero JE, Pagan A, Hellmuth C, Uhl O (2017). Placental MFSD2a transporter is related to decreased DHA in cord blood of women with treated gestational diabetes. Clin. Nutr. (Edinburgh, Scotland)..

[37] Toufaily C, Vargas A, Lemire M, Lafond J, Rassart E, Barbeau B (2013). MFSD2a, the Syncytin-2 receptor, is important for trophoblast fusion. Placenta.

[38] Madeleneau D, Buffat C, Mondon F, Grimault H, Rigourd V, Tsatsaris V (2015). Transcriptomic analysis of human placenta in intrauterine growth restriction. Pediatr. Res..

[39] Hu M, Li J, Baker PN, Tong C (2021). Revisiting preeclampsia: A metabolic disorder of the placenta. FEBS J..

[40] Khaire AA, Thakar SR, Wagh GN, Joshi SR (2021). Placental lipid metabolism in preeclampsia. J. Hypertens..

[41] Vluggens A, Andreoletti P, Viswakarma N, Jia Y, Matsumoto K, Kulik W (2010). Reversal of mouse Acyl-CoA oxidase 1 (ACOX1) null phenotype by human ACOX1b isoform [corrected]. Lab. Invest..

[42] Zeng J, Deng S, Wang Y, Li P, Tang L, Pang Y (2017). Specific inhibition of acyl-CoA oxidase-1 by an acetylenic acid improves hepatic lipid and reactive oxygen species (ROS) metabolism in rats fed a high fat diet. J. Biol. Chem..

[43] Huang J, Jia Y, Fu T, Viswakarma N, Bai L, Rao MS (2012). Sustained activation of PPARα by endogenous ligands increases hepatic fatty acid oxidation and prevents obesity in ob/ob mice. FASEB J..

[44] Burri L, Thoresen GH, Berge RK (2010). The role of PPARα activation in liver and muscle. PPAR Res..

[45] Bougarne N, Weyers B, Desmet SJ, Deckers J, Ray DW, Staels B (2018). Molecular actions of PPARα in lipid metabolism and inflammation. Endocr. Rev..

[46] Shearer BG, Steger DJ, Way JM, Stanley TB, Lobe DC, Grillot DA (2008). Identification and characterization of a selective peroxisome proliferator-activated receptor beta/delta (NR1C2) antagonist. Mol. Endocrinol..

[47] Hebbachi AM, Knight BL, Wiggins D, Patel DD, Gibbons GF (2008). Peroxisome proliferator-activated receptor alpha deficiency abolishes the response of lipogenic gene expression to re-feeding: Restoration of the normal response by activation of liver X receptor alpha. J. Biol. Chem..

[48] Miller CW, Ntambi JM (1996). Peroxisome proliferators induce mouse liver stearoyl-CoA desaturase 1 gene expression. Proc. Natl. Acad. Sci. USA.

[49] Talbot CPJ, Dolinsky VW (2019). Sex differences in the developmental origins of cardiometabolic disease following exposure to maternal obesity and gestational diabetes. Appl. Physiol. Nutr. Metab..

[50] Dearden L, Bouret SG, Ozanne SE (2018). Sex and gender differences in developmental programming of metabolism. Mol. Metab..

[51] Evans L, Myatt L (2017). Sexual dimorphism in the effect of maternal obesity on antioxidant defense mechanisms in the human placenta. Placenta.

[52] Brass E, Hanson E, O'Tierney-Ginn PF (2013). Placental oleic acid uptake is lower in male offspring of obese women. Placenta.

[53] Al-Qaraghouli M, Fang YMV (2017). Effect of fetal sex on maternal and obstetric outcomes. Front. Pediatr..

[54] Shook LL, Kislal S, Edlow AG (2020). Fetal brain and placental programming in maternal obesity: A review of human and animal model studies. Prenat. Diagn..

[55] Shrestha N, Ezechukwu HC, Holland OJ, Hryciw DH (2020). Developmental programming of peripheral diseases in offspring exposed to maternal obesity during pregnancy. Am. J. Physiol. Regul. Integr. Comp. Physiol..

[56] Sun T, Gonzalez TL, Deng N, DiPentino R, Clark EL, Lee B (2020). Sexually dimorphic crosstalk at the maternal-fetal interface. J. Clin. Endocrinol. Metab..

[57] Eriksson JG, Kajantie E, Osmond C, Thornburg K, Barker DJ (2010). Boys live dangerously in the womb. Am. J. Hum. Biol..

[58] Lassance L, Haghiac M, Minium J, Catalano P, Hauguel-de MS (2015). Obesity-induced down-regulation of the mitochondrial translocator protein (TSPO) impairs placental steroid production. J. Clin. Endocrinol. Metab..

