# Supplementary material for: Obesity downregulates lipid metabolism genes in first trimester placenta
Source: Sci Rep. 2022 Nov 12;12:19368. doi: 10.1038/s41598-022-24040-9 (PMC9653480; doi:10.1038/s41598-022-24040-9)
Supplement: Supplementary file 1 — Supplementary Information. [file 41598_2022_24040_MOESM1_ESM.pdf]

Obesity downregulates lipid metabolism genes in first trimester placenta

Aisha Rasool[1], Taysir Mahmoud[1], Begum Mathyk[2], Tomoko Kaneko-Tarui[1], Danielle Roncari[3], Katharine O. White[4], Perrie O'Tierney-Ginn*[1,3,5]

[1]Mother Infant Research Institute, Tufts Medical Center, Boston, MA; [2]Brandon Regional Hospital, Brandon, FL; [3]Dept Obstetrics & Gynecology, Tufts University School of Medicine; [4]Dept Obstetrics & Gynecology, Boston University School of Medicine; [5]Friedman School of Nutrition, Tufts University, Boston, MA

Supplementary Table 1

Supplementary Data for Fig.1

	Gene	Gene Symbol	Log2 Fold Change	Log2 Std Error	P Value	Q Value
**Peroxisomal Oxidation**	ACOX1	ACOX1	-0.342	0.0626	1.14E-05	0.00039
	PEX3	PEX3	-0.146	0.115	0.22	0.4
	COT	CROT	-0.215	0.199	0.29	0.5
**Mitochondrial FA Oxidation**	CPT2	CPT2	-0.534	0.126	0.00026	0.0045
	AMPKa	PRKAA1	-0.263	0.08	0.0032	0.018
	OCTN2	SLC22A5	-0.249	0.13	0.067	0.16
	COX-7b	COX7B	-0.059	0.057	0.32	0.52
	CACT	CACT	0.113	0.119	0.35	0.53
	ACAT-1	ACAT1	-0.108	0.159	0.5	0.6
	ACAA2	ACAA2	0.04	0.103	0.7	0.8
	PPARα	PPARA	0.0197	0.105	0.85	0.8
	CPT1b	CPT1B	-0.03	0.183	0.87	0.86
**Lipases**	LPL	LPL	-1.31	0.336	0.0006	0.007
	EL	LIPG	-0.356	0.126	0.009	0.039
**FA Uptake and Transporters**	MFSD2A	MFSD2A	-0.625	0.178	0.0017	0.012
	FATP4	SLC27A4	-0.256	0.106	0.023	0.086
	FATP3	SLC27A3	-0.219	0.092	0.025	0.086
	CD36	CD36	-0.562	0.268	0.046	0.1
	FABPpm	GOT2	0.117	0.076	0.14	0.3
	FABP4	FABP4	0.643	0.445	0.16	0.3
	VLDL-R	VLDLR	-0.193	0.133	0.16	0.3
	FATP6	SLC27A6	-0.183	0.218	0.41	0.5
	FABP3	FABP3	-0.12	0.154	0.44	0.6
	LDL-R	LDLR	0.03	0.077	0.7	0.68
	FATP2	SLC27A2	0.043	0.166	0.8	0.8
	FABP5	FABP5	0.009	0.106	0.9	0.86
**Lipogenesis**	DGAT1	DGAT1	-0.357	0.082	0.0002	0.003
	PLIN2	PLIN2	-0.877	0.247	0.0016	0.012
	ACC	ACACA	-0.256	0.082	0.0044	0.021
	ACSS2	ACSS2	-0.256	0.111	0.03	0.09
	ACSL1	ACSL1	-0.556	0.256	0.04	0.1
	ACSL5	ACSL5	-0.372	0.498	0.46	0.6
	SREBP1	SREBF1	-0.131	0.11	0.24	0.4
	SCD-1	SCD	0.097	0.162	0.56	0.64
	PPARγ	PPARG	-0.018	0.119	0.88	0.86

Supplementary Table 2 *Supplementary data for Figure 4*

Maternal	(n=7)
**Age, y**	26.7 ± 5.3
**BMI, kg/m²**	26 ± 5.6
**Gestational Age**	11.4 ± 2.7
**Smoker (N:Y)**	3:4
**Race**	2 Caucasian<br>4 African American<br>1 Hispanic
**Cholesterol, mU/ml**	170 ± 41.4
**Triglycerides, mg/dl**	81.9 ± 22.9
**Glucose, mg/dl**	81.3 ± 7.2
**Insulin, mU/ml**	±

Supplementary Table 3

Supplementary Data for Sex Graphs

Maternal BMI Group	Fetal Sex	Number	BMI	GA	Mat Age	Smokers/Non-Smokers	Race
**Lean**	Male	10	21.5 $\pm$ 2.0	9.9 $\pm$ 1.9	25.1 $\pm$ 5.7	2/8	6C,1M, 1AA, 1H, 1A
**Obese**	Male	5	39.4 $\pm$ 9.2	9.1 $\pm$ 1.1	29.4 $\pm$ 4.9	2/3	3C, 2AA
**Lean**	Female	4	20.5 $\pm$ 1.8	9.3 $\pm$ 1.2	28.5 $\pm$ 4.7	2/2	3C, 1M
**Obese**	Female	8	33.6 $\pm$ 2.9	9.6 $\pm$ 1.4	29.4 $\pm$ 8.6	1/6	2C, 4AA, 1H, 1N/A

C, Caucasian; AA, African-American; H, Hispanic; M, Mixed Race; N/A, no race recorded. Values are means ± SDs. $P < 0.05$. Mann Whitney Tests were used for all except smokers and race where chi-squared test was used.

Supplemental Figure

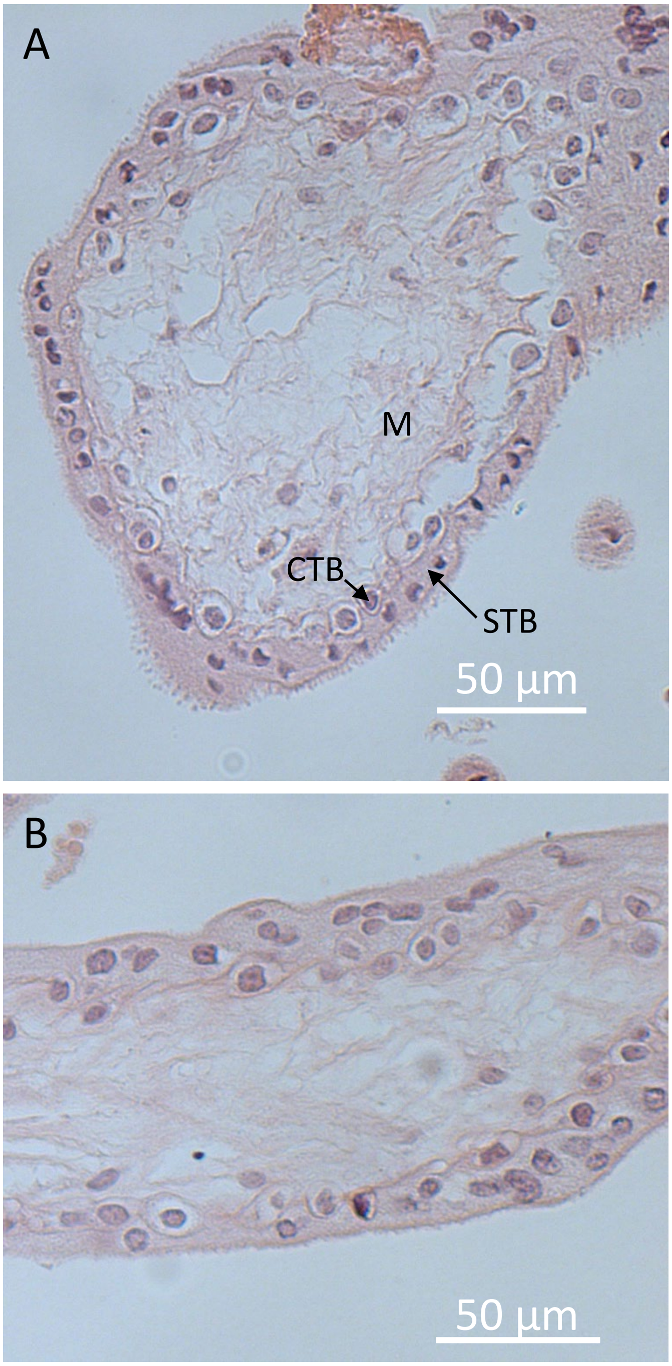

Supplemental Figure. Representative micrographs of first trimester placenta following fixation in Zinc-based fixative, embedding, and H&E staining. A: Micrograph of placental villi at 10 weeks gestation from a woman with BMI=20 kg/m2. B: Micrograph of placental villi at 9.5 weeks gestation from a woman with BMI=37 kg/m2. Microvilli brush border clearly visible on surface of syncytiotrophoblast layer. M=mesenchyme; CTB=cytotrophoblast; STB=syncytiotrophoblast layer
